# Mental health impact among hospital staff in the aftermath of the Nice 2016 terror attack: the *ECHOS de Nice* study

**DOI:** 10.1186/s12889-021-11438-9

**Published:** 2021-07-10

**Authors:** Laurence Bentz, Stéphanie Vandentorren, Roxane Fabre, Jeremy Bride, Philippe Pirard, Nadège Doulet, Thierry Baubet, Yvon Motreff, Christian Pradier

**Affiliations:** 1 Côte d’Azur University, Nice University Hospital, Public Health Department, Nice, France; 2 Santé publique France, French national public health agency, Direction scientifique et internationale, F-94415 Saint-Maurice, France; 3 Université Bordeaux, INSERM UMR 1219, Vintage team, F-33000 Bordeaux, France; 4 Côte d’Azur University, CoBTeK lab, Nice University Hospital, CMRR, Nice, France; 5 Côte d’Azur University, Nice University Hospital, Policlinique, Medical and psychological emergency unit (CUMP 06), Nice, France; 6 Paris 13 Sorbonne University - Paris Cité, Laboratoire UTRPP (EA 4403), Villetaneuse, France; 7 Santé publique France, French national public health agency, Non-Communicable Diseases and Trauma Division, F-94415 Saint-Maurice, France; 8 Team MOODS, CESP, Inserm 1178, Paris-Saclay University, UVSQ, Villejuif, France; 9 Centre National de Ressources et de Résilience (CN2R), Lille/Paris, France; 10 UTRPP ER 4403, Sorbonne Paris Nord University, Villetaneuse, France; 11 Assistance Publique - Hôpitaux de Paris, Avicenne Hospital, Child and adolescent psychopathology, psychiatry and addiction, Bobigny, France; 12 Sorbonne Université, Inserm, Institut Pierre Louis d’Épidémiologie et de Santé Publique (IPLESP), Department of Social Epidemiology, F-75012 Paris, France

**Keywords:** Terror attack, Hospital staff, Healthcare workers, Stress disorders, Post-traumatic stress, Healthcare use, Occupational health

## Abstract

**Background:**

The Nice terror attack of July 14, 2016 resulted in 84 deaths and 434 injured, with many hospital staff exposed to the attack, either as bystanders on site at the time of the attack (‘bystander exposure’) who may or may not have provided care to attack victims subsequently, or as care providers to victims only (‘professional exposure only’). The objective of this study is to describe the impact on mental health among hospital staff by category of exposure with a particular focus on those with ‘professional exposure only’, and to assess their use of psychological support resources.

**Method:**

An observational, cross-sectional, multicenter study conducted from 06/20/2017 to 10/31/2017 among all staff of two healthcare institutions in Nice, using a web questionnaire. Collected data included social, demographic and professional characteristics; trauma exposure category (‘bystanders to the attack’; ‘professional exposure only’; ‘unexposed’); indicators of psychological impact (Hospital Anxiety and Depression Scale); PTSD (PCL-5) level; support sought. Responders could enter open comments in each section of the questionnaire, which were processed by inductive analysis.

**Results:**

804 staff members’ questionnaires were analysed. Among responding staff, 488 were exposed (61%): 203 were ‘bystanders to the attack’, 285 had ‘professional exposure only’. The staff with ‘professional exposure only’ reported anxiety (13.2%), depression (4.6%), suicidal thoughts (5.5%); rates of full PTSD was 9.4% and of partial PTSD, 17.7%. Multivariate analysis in the ‘professional exposure only’ category showed that the following characteristics were associated with full or partial PTSD: female gender (OR = 2.79; 95% CI = 1.19–6.56, *p* = 0.019); social isolation (OR = 3.80; 95% CI = 1.30–11.16, *p* = 0.015); having been confronted with an unfamiliar task (OR = 3.04; 95% CI = 1.18–7.85; *p* = 0.022). Lastly, 70.6% of the staff with ‘professional exposure only’ with full PTSD did not seek psychological support.

**Conclusion:**

Despite a significant impact on mental health, few staff with ‘professional exposure only’ sought psychological support. Robust prevention and follow-up programs must be developed for hospital staff, in order to manage the health hazards they face when exposed to exceptional health-related events such as mass terror attacks.

**Study registration:**

Ethical approval for the trial was obtained from the National Ethics Committee for Human Research (RCBID N° 2017-A00812–51).

## Background

Health care systems now face the challenge of a surge of terror attacks across Europe over the past ten years. Aside from the significant number of victims either dead or injured, these attacks generate psychological trauma in the exposed general population and in care providers.

Post-traumatic stress disorders (PTSD) have been described among emergency department professionals [1, 2], along with depression, anxiety [3], suicidal thoughts [4], alcohol, drug or medication abuse or addiction [5] and occurrence of comorbidities [6]. Whether PTSD is full or partial [7, 8], the resulting functional disorders are detrimental to all aspects of life, including professional activity [9]. Several studies conducted worldwide [2, 10], and in Europe, have described the consequences of the terror attacks in Oslo and Utoya, Madrid [11], London [12], and Berlin [13]. Similar mental health outcomes have been observed in France among care providers following the 2015 Paris attacks [1, 14–16], highlighting the need to provide these professionals with therapeutic assistance.

Soon after the Paris attacks, the city of Nice was the scene of a terror attack on July 14, 2016. A truck rammed the crowd along nearly 2 km during the Bastille day fireworks display, resulting in 86 dead, among whom 10 were children or adolescents, and 434 injured. Among the tens of thousands who had come to watch the fireworks, healthcare professionals were also present with their families, outside their professional scope.

An emergency plan was set up to organise the response by healthcare institutions and manage a massive influx of victims: most of the adults were directed towards Nice University Hospitals (Centre Hospitalo-Universitaire de Nice, CHU), and children and adolescents towards the CHU and Lenval Foundation hospitals. Following immediate on-site intervention by first responders, the entirety of the hospital staff of the 6 CHU and Lenval institutions were mobilized over several weeks to support to patient care. The consequences of such professional exposure may have been harmful to workers’ health, due to the large number of victims, their distribution and concentration within the two Nice institutions in the context of complex crisis-related logistics, the involvement of a paediatric population, and the highly damaging injuries resulting from the truck’s crushing effect [17–20].

Some members of the hospital staff may have been doubly exposed, both privately and professionally. The effect on the overall staff’s mental health has rarely been described in such a context of exceptional mobilization. While studies have focused on the psycho-trauma sustained by first-line rescue workers, data are scarce regarding hospital staff involved in the general running of the institution. However, for hospitals involved in managing populations exposed to terror attacks, documenting the consequences of such an event on the health of all staff members appears essential to implement appropriate measures [21].

In France, collective emergency measures are deployed in the aftermath of an attack, including Medical-psychological emergency units: in Nice, psychiatrists, psychologists and volunteers intervened to ensure immediate and post-immediate care of the victims, the healthcare providers and rescuers on site, as well as members of hospital staff who expressed the need for it [22]. Under the aegis of Nice hospital management, mobile teams including psychiatrists and psychologists were set up to reach out to the most exposed participants; an anonymous psychological support helpline exclusively dedicated to staff was set up in each hospital site for 6 months; the occupational health departments of the CHU and Lenval hospitals contributed in receiving and directing hospital staff towards specialized care departments according to each one’s particular situation. A specific care pathway was established for medical students. Lastly, 1 year after the attack, since submission of the web questionnaire could result in the recall of traumatic experiences by some of the respondents, a new support facility was offered, i.e. availability of a clinical psychologist who could contact respondents wishing to provide their details and eventually organize appointments in view of specialized care (within the hospital or in a private medical practice). However, the impact of these resources on exposed staff is difficult to assess.

The aim of this study is to.

-describe the mental health impact (depression, anxiety, suicidal thoughts, full or partial PTSD, somatic symptoms) among University and Lenval Hospitals staff members by category of exposure with a particular focus on those with ‘professional exposure only’,

-assess the social, demographic and professional factors and the type of exposure related to the occurrence of full or partial PTSD,

- describe the use of mental health support resources by the exposed healthcare professionals.

## Method

### Design and study population

This was a cross-sectional, multi-centre, observational study. The detailed description of the study protocol has been described elsewhere [23].

The study was approved by the French Data Protection Authority and the Ethics Committee in accordance with the Declaration of Helsinki and registered under N° ID RCB: 2017-A00812–51.

The study focused on all hospital staff and students above 18 years of age registered with Nice university and Lenval hospitals from July 13, 2016, until the date of the study (conducted between June and October 2017). Overall, 10,100 subjects were registered on the Human Resources Departments’ lists of both hospitals in June 2017.

These subjects were offered to complete a web questionnaire with the help of the institutions’ Human Resources Departments. A letter was attached to the payslip of each staff member in June 2017. Information regarding the objectives and the procedures related to the survey were available on the intranet sites of both institutions. Specific communication activities were conducted among the teams of several departments and staff representatives. Staff that were not or no longer employed in the institutions between July 2016 and the beginning of the study were not included.

Access to the website also facilitated access to information on psychological trauma and its determinants.

### Exposure groups

We defined three main exposure categories for the purpose of this study, categorized as follows:

- Staff who were on site during the attack in a private capacity (‘bystander exposure’) with two further subgroups:
  - Staff who did not participate in the care for victims then or later ‘bystander exposure only’;
  - Staff who did participate in the care of attack victims ‘mixed exposure’;
- Staff who were not on site during the attack but cared for victims either as first responders or later (‘professional exposure only’);
- Unexposed staff members (exposed neither as bystanders on the attack site nor through their professional activity in relation to the attack) (‘Unexposed’).

Our study focuses mainly on the mental health impact among the staff with ‘professional exposure only’ because to our knowledge few studies have focused on those exposed exclusively through their professional duties.

### Non-inclusion criteria

Those below 18 years of age when completing the web-questionnaire; those not or no longer employed by the institutions concerned as: hospital staff; medical students and residents; paramedical students, between July 2016 and the beginning of the study were not included in the study.

### Data collection

#### Web-based questionnaire

Cross-functional working groups were involved in developing the web questionnaire to adapt its contents to the situation in Nice healthcare institutions and take staff members’ experience and suggestions into account. Once the web questionnaire was available online, staff members could read the information describing the survey and obtain an access code via their mobile phone if they chose to participate and therefore provide their phone number. For each person who connected, the eligibility was verified via the online inclusion questionnaire. In the following part of the web questionnaire, informed consent was obtained from all participants. Each respondent then accessed the rest of the survey: according to the various types of exposure experienced, filter questions guided respondents towards the corresponding paths. Completion of the questionnaire was estimated to require 20 to 45 min according to the type of exposure. The questionnaire was to be completed between June 21 and October 30, 2017, i.e. 11 to 15 months after the attack.

#### Information collected

We collected socio-demographic (age, gender, educational level, marital status) and occupational information (namely: adult or pediatric institution; profession; type of tasks performed). Regarding exposure, circumstances and degree of trauma exposure were assessed. For those members of staff who were professionally exposed, the tasks they performed were grouped into three categories:

- Tasks focusing on victims’ bodies, whether living or deceased (clinical, surgical, forensic, but also transport of living or deceased victims, imaging, cleaning…),

- Tasks related to the psychological support of victims and their families; death announcements to the family; communication and support of persons in distress (providing comfort, translating for foreign nationals); supporting distressed staff and students,

- Tasks related to crisis management (setting up of an emergency plan), call processing platform for emergency services, management of operational hospital reinforcement personnel (technical, logistical), identification and follow-up of victims throughout their care pathway, administrative (patient file completion and updating, etc.…) or logistical tasks.

The following Indicators of psychological and psycho-traumatic impact were assessed:

- The presence of probable full or partial PTSD was assessed on the basis of answers to the PTSD Checklist for DSM-5 (PCL-5) [24] which is a scale assessing both presence and intensity (0–4) of the 20 symptoms divided into 4 categories (criteria B to E). Each item of the PCL5 with a rating of 2 was defined as a PTSD symptom. The *DSM-5* diagnostic rule for full PTSD requires 4 of these DSM-5 criteria: at least 1 B item (questions 1 to 5), 1 C item (questions 6 to 7), 2 D items (questions 8 to 14), 2 E items (questions 15 to 20). For partial PTSD, we used the McLaughlin’s definition of this condition, which was defined as meeting 2 or 3 of DSM-5 Criteria B, C, D or E [7]. Respondents were to present with these symptoms during the month before completing the questionnaire, and the symptoms had to be associated with the traumatic event (exposure on the attack site and/or in the context of a professional activity in relation to the attack). Due to the DSM-5 requirement of exposure to a traumatic event to define possible PTSD, a filter was applied to the web questionnaire so that only staff members directly or indirectly exposed to the attack could complete the PCL5. The PTSD score was thus not computed for the unexposed group.

- Furthermore, potential functional disorders (difficulties with family, friends, work or everyday life) were investigated.

- Symptoms of anxiety and depression were assessed using the Hospital Anxiety and Depression Scale (HADS). This scale measures the intensity of perception of seven symptoms indicative of anxiety (7 questions scoring 0 to 3) and of seven symptoms indicative of depression (7 questions scoring 0 to 3) during the previous week. A score between 8 and 10 was considered as a possible state of anxiety or depression; a score above 11 as a probable one that should call for specialist assessment [25, 26].

- The occurrence of suicidal thoughts was explored, starting from the date of the attack.

- Changes after the terror attack in tobacco, alcohol and marijuana use, changes in medication (for sleep disturbances, fear or stress, depression) were also investigated.

- Thirteen questions explored the somatic impact.

- Occupational accident notification or work stoppage prescriptions were investigated.

- Questions explored the awareness of specialised support resources provided in the context of the survey, and care follow-up.

Lastly, staff were asked whether they had received psychological support in the aftermath of the attack, within the institution or elsewhere, regardless of the delay, the type of support (psychiatrist, psychologist, occupational physician, general practitioner, etc.…) and whether there had been a follow-up, as the survey aimed to determine the proportion of staff members who accessed psychological support and care follow-up.

### Analysis

#### Statistical analysis

Quantitative variables were described using mean and standard deviation, and qualitative variables were described using frequency and percentage.

Regarding the impact of professional exposure, the psycho-traumatic impact of the attack measured with the PCL5 and its the consequences on professional activity were compared among two groups: the ‘professional exposure only’ group and the ‘bystander exposure” group.

Regarding the mental health impact (anxiety, depression, suicidal thoughts, alcohol, tobacco, self-medication and specific treatments, somatic disorders), three groups were compared: the two groups of exposed staff (‘professional exposure only’ and ‘bystander exposure”) were compared to the unexposed staff.

Comparisons between the two groups (‘professional exposure only’; ‘bystander exposure’) were performed using Student’s t-test for quantitative variables and the Chi2 test or Fisher’s exact test (when expected frequencies were below 5) for qualitative variables. Comparisons between the three groups (‘professional exposure only’; ‘bystander exposure’; ‘unexposed’) were performed using ANOVA for quantitative variables and the Chi2 test for qualitative variables. A *p*-value < 0.05 was considered significant.

To assess factors associated with full or partial PTSD, a multivariate analysis was performed using logistic regression. The outcome variable fell into 2 modalities: full or partial PTSD, and neither full PTSD nor partial PTSD, with the latter chosen as reference. Based on published data [15], gender, age, marital status, social isolation, educational level, adult vs pediatric institution, profession and professional tasks were tested. Factors with a *p-*value < 0.10 in univariate analysis were included in the multivariate model. Factors with a *p-*value < 0.05 were kept in the final model. Statistical analyses were performed using the SAS Enterprise Guide 7.1 software®.

#### Qualitative analysis

Open comments allowed participants to describe their experience: mode of exposure during the night of the attack and subsequently; consequences on their professional, social and family life; psychological impact; use and perceived psychological assistance and social support.

To analyse respondents’ open comments, the general inductive approach for qualitative data analysis was used [27, 28]. Two researchers compared the results of their analyses to identify the emerging main or significant themes from emerging from the raw data. According to these themes, verbatim quotes were chosen to illustrate the quantitative results. The wording has been kept as expressed by the respondents; however, spelling was corrected in the transcript.

## Results

### Participation and respondents socio-demographic profiles

**Fig. 1:**
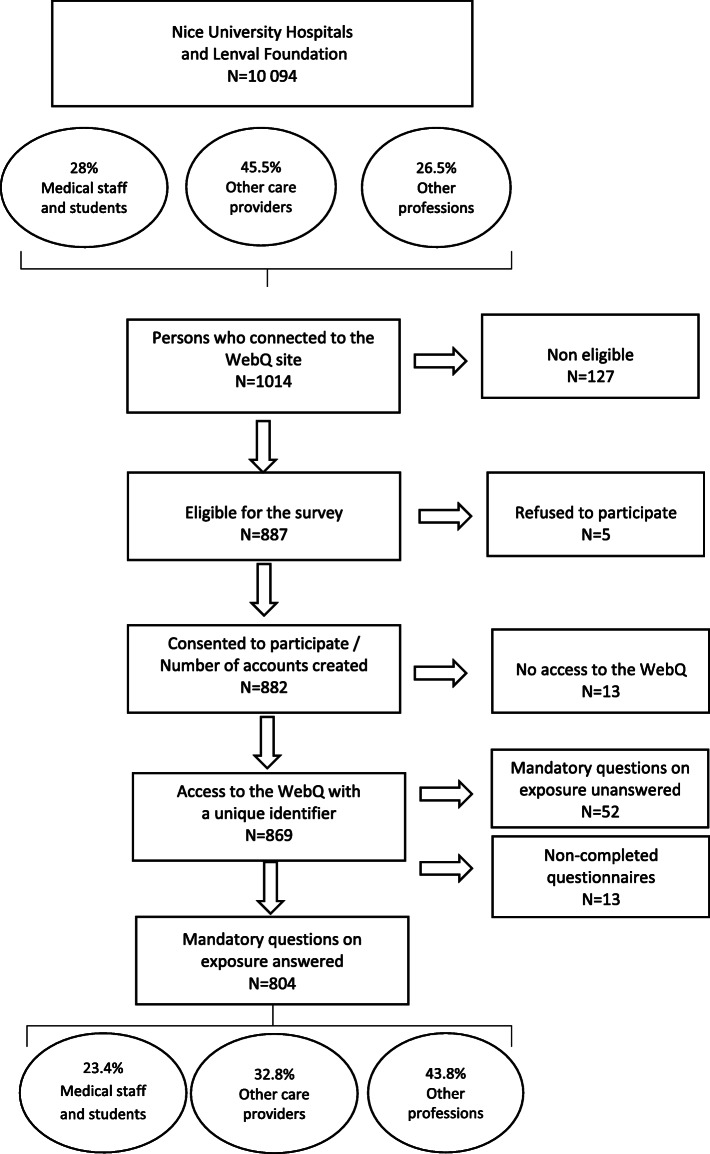
Flowchart

A flowchart of the study population is shown in Fig. 1. Finally, 804 staff members were eligible for analysis.

The distribution of respondents differs from that of the entire staff (*p* < 0.01) with fewer medical staff, (23.4% vs 28%) and other care providers (32.8% vs 45.5%); in contrast, the study population includes a larger proportion of “other professionals” i.e. those in charge of managerial, administrative and technical activities e.g. IT professionals (43.8% vs 26.5%,).

Non-medical CHU personnel accounted for more than three quarters of our study population. Compared to non-medical CHU personnel, this sample included more female respondents (79.1% vs 73.0%, *p* = 0.002), staff members aged 30–50 years (32.7% vs 26.1%, *p* < 0.001), management and administrative staff, and technical staff (32.5% vs 13.2, 20.1% vs 5.9%, respectively, *p* < 0.001).

Most respondents (86%) worked in one of the Nice university hospitals that care for adults, while 14% were members of the Lenval paediatric hospital staff.

**Table 1:** Professional tasks performed by the staff with ‘professional exposure only’ and by the staff with ‘bystander exposure’

	Professional exposure only (***n*** = 285)		Bystander exposure (***n*** = 203)		*p*-value
	N^a^	(%)	N^a^	(%)	
**Hospital specialty**					0.194
Adult departments	223	(78.6)	169	(82.2)	
Pediatric departments	61	(21.4)	34	(16.8)	
**Performed tasks involving physical care**					<.001
Yes	222	(77.9)	88	(43.4)	
No	63	(22.1)	115	(56.7)	
**Performed tasks involving deceased bodies**					0.383
Yes	68	(24.3)	42	(20.9)	
No	212	(75.7)	159	(79.1)	
**Performed tasks involving injured or deceased children**					0.002
Yes	114	(40.9)	55	(27.4)	
No	165	(59.1)	146	(72.6)	
**Provided psychological support to victims or families / Had to communicate with families**					<.001
Yes	233	(81.8)	88	(43.4)	
No	52	(18.3)	115	(56.6)	
**Performed administrative tasks**					<.001
Yes	176	(61.8)	63	(31.0)	
No	109	(38.3)	140	(69.0)	
**Were confronted with such tasks for the first time**					<.001
Yes	214	(77.0)	87	(43.1)	
No	64	(23.0)	115	(56.9)	
**Performed tasks unrelated to their training**					0.001
Yes	83	(29.6)	32	(15.8)	
No	197	(70.4)	170	(84.2)	
	Mean	[SD]	Mean	[SD]	*p-*value
**Mean duration of professional exposure (hours) on July 14 and 15**	10.5	[±6.5]	3.0	[±5.4]	0.034
**Mean duration of professional exposure (days) from July 16**	22.1	[±26.3]	10.5	[±6.5]	<.001

^a^Cases

Moreover, respondents’ professional activity included 32.1% non-medical care-providers (nurses, health executives, psychologists, orderlies, physiotherapists, dieticians, stretcher bearers, ambulance staff, etc..); 23.3% administrative and managerial staff; 14.1% medico-technical staff; 13% medical staff, including surgeons and pharmacists.

### Exposure

**Fig. 2:**
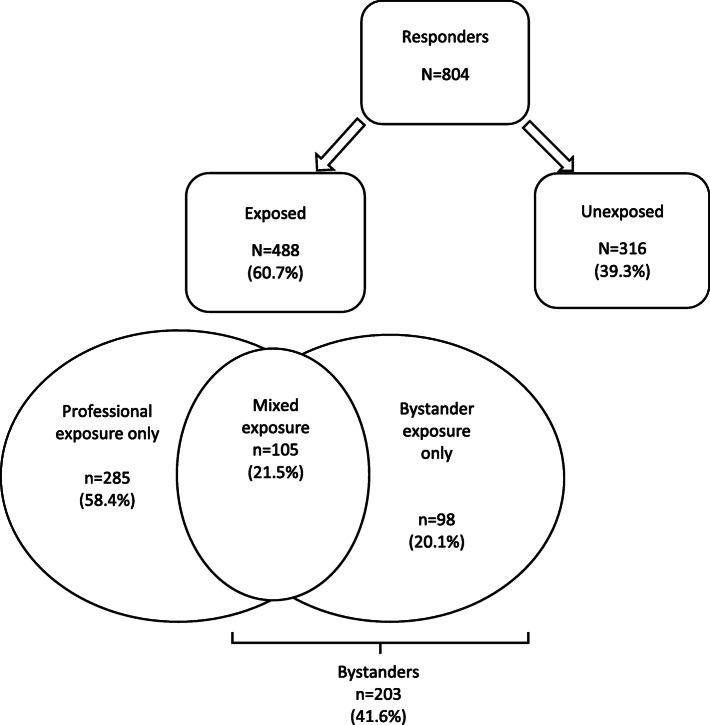
Exposure groups among responders (*N* = 804)

Figure 2 shows the distribution of type of exposure. Respondents who had been exposed numbered 488 (61.7%). Among these, the ‘professional exposure only’ category accounted for 58.4% (285/488) of respondents, and those in the ‘bystander exposure’ category for 41.6% (203/488). The remaining 39.3% of respondents were not exposed to the attack. Compared to all the hospital employees, the ‘professional exposure only’ staff, which is the focus of this study, differs in that it includes fewer other care providers (36.4% vs 45.5%) but more « other professions » (35.3% vs 26.5%). The proportion of medical professionals is however comparable (28.4% vs 28%).

Table 1 illustrate the professional tasks performed by the ‘professional exposure only’ staff which may be considered as aspects of indirect professional exposure**.** Almost 80% of ‘professional exposure only’ staff members performed tasks that put them in direct physical contact with victims, and almost one quarter with deceased bodies, whether of adults or children. Most of these ‘professional exposure only’ staff (78.6%) worked in adult departments while the remainder (21.4%) worked in paediatric departments, although almost twice that proportion performed tasks relating to injured or deceased children (40.9%). Lastly, almost 30% of ‘professional exposure only’ staff had to take on tasks outside the scope of their usual jobs and 77% had been faced with such tasks for the first time. The average duration of professional exposure of the ‘professional exposure only’ staff amounted to approximately 10 h from the night of July 14 and July 15, and an average of 20 days as from July 16.

#### Quotes pertaining to professional exposure

Emergency staff were assigned to the attack site under highly uncertain and alarming circumstances: *« A false announcement stated that the first-line emergency treatment unit had exploded, I thought I had killed my colleagues by sending them to the attack site. ».*

Comments revealed the exceptional intensity in terms of tasks and emotional impact of the hours of work in the immediate aftermath of the attack: « *A difficult moment, for the pressure had dropped and we realized at that moment what we had just been through».*

Among the comments revealing the particularly painful circumstances experienced within the work environment, several types of difficulties stood out: those relating to the management of child victims; to the search for and transmission of information concerning patients and their follow-up; the reception of families in a chaotic context: « *The overload. My colleagues’ faces totally devastated. All these people who were screaming, weeping. All these dead or suffering children. These distraught parents. » « To hear families wailing in distress »*.

Themes identified among the comments reveal stressful difficulties related to victim identification:

*-« The child which we cared for only had an identification number, certainly assigned by the emergency department… We had no name, parent nor address whatsoever for him. ».*

*-« Imaging procedures on numerous dislocated adult and child bodies ».*

### Effects on mental health

Impact on mental health among staff members is described in Table 2.

**Table 2:** Consequences on mental health

	Professional exposure only (***n =*** 285)		Bystanders (***n =*** 203)		Unexposed (***n*** = 316)		*p*-value
	N^a^	(%)	N^a^	(%)	N^a^	(%)	
**PTSD (PCL 5)**^**b**^							0.068
Probable Full PTSD	19	(9.4)	17	(12.2)	–	–	
Probable Partial PTSD	36	(17.7)	37	(26.6)	–	–	
No PTSD	148	(72.9)	85	(61.2)	–	–	
**HAD Anxiety scale**							0.611
> 10 (probable anxiety)	29	(13.2)	21	(14.7)	24	(10.3)	
8–10 (possible anxiety)	46	(21.0)	34	(23.8)	48	(20.7)	
≤ 7 (no anxiety symptoms)	144	(65.8)	88	(61.5)	160	(69.0)	
**HAD Depression scale**							0.848
> 10 (probable depression)	10	(4.6)	6	(4.1)	8	(3.5)	
8–10 (possible depression)	12	(5.5)	12	(8.2)	15	(6.5)	
≤ 7 (no depression symptoms)	196	(89.9)	129	(87.8)	209	(90.1)	
**Suicidal thoughts**							0.020
Present	12	(5.5)	9	(6.1)	3	(1.2)	
Absent	208	(94.5)	139	(93.9)	239	(98.8)	
**Tobacco**							<.001
Initiated or relapsed / Increased	33	(13.8)	26	(15.5)	13	(4.9)	
Other^c^	207	(86.2)	142	(84.5)	250	(95.1)	
**Alcohol intake**							0.085
Initiated or relapsed / Increased	11	(4.6)	13	(7.7)	8	(3.1)	
Other^c^	228	(95.4)	155	(92.3)	253	(96.9)	
**Self-medication**							0.116
Initiated or relapsed / Increased	17	(7.1)	14	(8.4)	10	(3.8)	
Other^c^	222	(92.9)	152	(91.6)	251	(96.2)	
**Since the attack, started taking medication for sleep disturbance**							0.003
Yes	8	(3.3)	12	(7.2)	1	(0.4)	
No	232	(96.7)	155	(92.8)	269	(99.6)	
**Since the attack, started taking medication for difficulties related to anxiety and/or stress**							0.001
Yes	9	(3.8)	11	(6.6)	1	(0.4)	
No	231	(96.2)	155	(93.4)	267	(99.6)	
**Since the attack, started taking medication for difficulties related to depression**							0.781
Yes	5	(2.1)	4	(2.4)	4	(1.5)	
No	234	(97.9)	164	(97.6)	265	(98.5)	

^a^Cases

^b^Finally, probable full or partial PTSD was 27.1% among staff with ‘professional exposure only’, and 38.8% among ‘bystanders’

^c^Other: discontinued consumption, consumption unchanged, never used or discontinued long ago

PTSD prevalence rates were higher among ‘bystanders’ compared to ‘professional exposure only’ subjects (full PTSD 12.2% vs 9.4%, and partial PTSD 26.6% vs 17.7%) although these differences did not reach statistical significance (*p* = 0.068). Finally, among all exposed staff members (*n* = 488), the prevalence rates of full or partial PTSD was 31.5% (10.5% for full PTSD, 21% for partial PTSD).

The degree of functional disorders was comparable among ‘professional exposure only’ staff and ‘bystanders’ (20.1% vs 23.6%, respectively *p* = 0.433). Functional impairments were more prevalent among staff with full or partial PTSD, with no significant difference between the two groups (41.5% vs 47.2% respectively, *p* = 0.56).

Both categories of exposed staff and that of the non-exposed staff were comparable regarding states of anxiety and depression.

However, both exposed groups had significantly higher rates of suicidal thoughts than those unexposed (5.5% among ‘professional exposure only’ staff, 6.1% for ‘bystanders and 1.2% for those unexposed; *p* = 0.02). More had started smoking again or increased their tobacco consumption (*p* < 0.001); their use of sleeping medications (*p* = 0.003); and of medications to help them cope with difficulties related to anxiety and stress (*p* = 0.001).

Factors associated with full or partial PTSD revealed by multivariate analysis among ‘professional exposure only’ staff members (203 respondents to the PCL5 questions) are shown in Table 3. Female gender (OR = 2.79; 95% CI = 1.19–6.56, *p* = 0.019), social isolation (OR = 3.80; 95% CI = 1.30–11.16, *p* = 0.015) and having been confronted with an ‘unfamiliar task’ (OR = 3.04; 95% CI = 1.18–7.85, *p* = 0.022) were associated with a higher risk for full or partial PTSD. Conversely, neither age, educational level, working institution, professional category nor type of task performed was associated with PTSD.

**Table 3:** Factors associated with full or partial PTSD among the ‘professional exposure only’ category (*N =* 203 respondents to the PCL5)

	Univariate analysis			Multivariate analysis		
	OR	[CI 95%]	*p*-value	Adj OR	[CI 95%]	*p*-value
**Gender**						
Male	1			1		
Female	2.91	[1.28; 6.63]	0.011	2.79	[1.19; 6.56]	0.019
**Age**						
< 30	1					
[30; 40[	0.93	[0.39; 2.22]	0.876			
[40; 50[	1.24	[0.53; 2.88]	0.618			
> =50	0.46	[0.18; 1.16]	0.101			
**Marital status**						
Lives with a partner	1					
Lives alone	1.53	[0.80; 2.90]	0.198			
**Social isolation**						
Yes (feels rather lonely / very lonely)	3.09	[1.16; 8.27]	0.025	3.80	[1.30; 11.16]	0.015
No (feels rather supported / well supported)	1			1		
**Educational level**						
Advanced education	1					
Secondary level education	1.43	[0.64; 3.19]	0.380			
**Hospital**						
Adult	1					
Paediatric	0.81	[0.37; 1.77]	0.591			
**Profession**						
Medical staff/care providers/students	1					
Other staff	1.54	[0.80; 2.93]	0.195			
**Awareness of psychological consequences**						
Yes	1					
No	2.54	[1.00; 6.44]	0.050			
**Performed tasks involving physical care**						
Yes	1.30	[0.57; 2.95]	0.531			
No	1					
**Performed tasks involving deceased bodies**						
Yes	0.98	[0.49; 1.98]	0.965			
No	1					
**Performed tasks involving injured or deceased children**						
Yes	1.24	[0.66; 2.32]	0.509			
No	1					
**Provided psychological support to, or communicated with families**						
Yes	1.20	[0.48; 2.98]	0.699			
No	1					
**Perfomed administrative tasks**						
Yes	1.07	[0.56; 2.02]	0.847			
No	1					
**Were confronted with this type of task for the first time**						
Yes	3.17	[1.26; 7.98]	0.014	3.04	[1.18; 7.85]	0.022
No	1			1		
**Performed tasks unrelated to their training**						
Yes	1.37	[0.71; 2.63]	0.347			
No	1					

#### Quotes related to symptoms pertaining to full or partial PTSD

Scenes were re-lived through various sensory reminders: « *What shocked me most: the smell of the corpses (…) with flashbacks when I breathed unpleasant smells for weeks following my work (…) for instance in the street if there was a strong smell, it immediately brought back the memory of corpses and what I had seen in the forensic department. ».*

Staff mentioned mood disturbances: *« More aggressive because on the defensive, I lock myself up in my bubble and I lose all motivation, everything is constrained »;* hypervigilance: *« Much more watchful in everyday life and in the hospital. In the operating theatre, paying even more attention to make sure the equipment is ready in case… ».* And, also, avoidance of situations that might bring back the traumatic experience: *« I don’t want to go out anymore. I prefer to see my friends in my home. ».*

### Physical effects

The physical impact since the attack according to exposure category is shown in Table 4. Prior to the attack, the two groups of exposed staff and the unexposed staff were comparable in terms of physical complaints. Following the attack, certain health issues arose or worsened among those exposed, with significantly more instances of back pain and osteoarticular complaints (*p* = 0.003), gastric pain/ulcers (*p* = 0.048), fatigue/weariness (*p* = 0.018), concentration difficulties (*p* < 0.001), and sleep disorders (*p* < 0.001).

**Table 4:** Somatic impact since the attack according to exposure group

Symptoms and conditions arisen since the attack	Professional exposure only (***n*** = 285)		Bystanders (***n*** = 203)		Unexposed (***n*** = 316)		*p*-value
	N^a^	(%)	N^a^	(%)	N^a^	(%)	
**Headaches, migraine**							0.174
Yes	122	(52.6)	75	(47.5)	113	(44.1)	
No	110	(47.4)	83	(52.5)	143	(55.9)	
**Back pain – joint pain**							0.003
Yes	123	(53.0)	85	(54.1)	102	(39.8)	
No	109	(47.0)	72	(45.9)	154	(60.2)	
**Abdominal pain, colic**							0.435
Yes	69	(29.9)	48	(30.4)	64	(25.4)	
No	162	(70.1)	110	(69.6)	188	(74.6)	
**Asthma – other respiratory issues**							0.678
Yes	13	(5.9)	7	(4.7)	17	(6.9)	
No	208	(94.1)	142	(95.3)	231	(93.2)	
**Stomach pain – Gastric ulcer**							0.048
Yes	56	(24.4)	32	(20.5)	39	(15.4)	
No	174	(75.6)	124	(79.5)	214	(84.6)	
**Arterial hypertension**							0.089
Yes	20	(8.7)	9	(5.7)	10	(4.0)	
No	209	(91.3)	148	(94.3)	243	(96.0)	
**Skin conditions**							0.112
Yes	44	(18.9)	36	(23.5)	38	(15.2)	
No	189	(81.1)	117	(76.5)	212	(84.8)	
**Uncontrolled diabetes**							0.641
Yes	1	(0.4)	2	(1.3)	2	(0.8)	
No	229	(99.6)	151	(98.7)	246	(99.2)	
**Heart trouble**							0.5
Yes	7	(3.0)	4	(2.6)	4	(1.6)	
No	223	(97.0)	151	(97.4)	247	(98.4)	
**Fatigue / Weariness**							0.018
Yes	129	(56.1)	97	(61.4)	120	(47.6)	
No	101	(43.9)	61	(38.6)	132	(52.4)	
**Difficulty to concentrate**							<.001
Yes	55	(23.6)	61	(39.4)	45	(17.8)	
No	178	(76.4)	94	(60.6)	208	(82.2)	
**Sleep disorder**							<.001
Yes	105	(44.9)	88	(57.1)	91	(36.2)	
No	129	(55.1)	66	(42.9)	160	(63.8)	
**Tinnitus**							0.602
Yes	17	(7.4)	16	(10.3)	22	(8.8)	
No	213	(92.6)	139	(89.7)	229	(91.2)	
**Unintentional weight change since the attack**							0.125
Yes (+/− 10%)	29	(12.0)	27	(16.1)	27	(10.2)	
No	189	(78.5)	120	(71.4)	219	(82.3)	
Don’t know	23	(9.5)	21	(12.5)	20	(7.5)	

^a^Cases

#### Quotes: Health issues with impact on work

Certain comments confirm health issues.

*-« Heightened emotivity; decreased ability to cope with emotions; recurring low back pain in November December, while I had never stopped work before».*

*-« I was psychologically crippled, I couldn’t run nor go to work on foot,* (because of) *extreme weariness linked to insomnia».*

Other comments relate to changes in eating habits:

*«I pay less attention to what I eat because I tell myself that there’s no point in depriving myself since I may not be here tomorrow».*

*-« Since I was very disturbed by the sight of corpses and the smell of blood, I’ve changed my eating habits and don’t eat meat anymore. ».*

### Professional repercussions

Concerning the professional sequellae, 12.9% of exposed respondents (*N* = 488) were relieved from their position following their involvement in the immediate aftermath of the attack; 8.9% were prescribed sick leave in relation to the event; 1.6% were declared victims of an occupational accident. No statistically significant difference was observed between the groups, aside from occupational accidents (1.2% among ‘professional exposure only’ staff vs 2.2% among ‘bystanders, *p* = 0.032).

#### Quotes pertaining to the consequences of the experience on professional activity

Quotes illustrate the types of impact on the ability to carry on with work:

*-« I had to ask urgently for a dozen days’ leave to stay with a family member who was in intensive care».*

*-« Unable to carry on with any or my own work. (…) I have almost no contact with my work colleagues, I just can’t. I still can’t go back* (to my workplace) *where I’ve been working enthusiastically for almost 15 years. ».*

Long-term consequences are still felt at the time of the survey:

*-« There’s a before and an after July 14th. I can’t manage to get involved in my work, many of the problems voiced by my colleagues seem childish; working conditions are becoming more wearisome and I wish to focus on my family. ».*

*-« For the time being I’ve lost my passion: my job. I have huge financial problems (…). I’m usually a very cheerful person; My joyfulness, I lost that on the night of July 14th, 2016. ».*

*-« I often think about it. Was I able to provide support to my team? I’m afraid it might happen again. And if so, how? ».*

Other comments, however, relate to the lessons learned from the experience:

*-« The problem of the terror attack, even though I was not personally involved, made me aware of the problems of work-related suffering and psycho-trauma ».*

Perceived changes are also linked to those felt within the hospital atmosphere and interpersonal relationships. Some are perceived as detrimental:

*- « THE ATMOSPHERE OF THE CITY AND THE CHU, WE WERE ALL DISORIENTED AND SHOCKED, OUR GAZE STOOD FOR WORDS. ».*

While others draw positive changes from this event:

*-« This experience hasn’t changed me, but I would say that it reinforced my pride in being a nurse while knowing that we, as an institution, only did our duty as a public service. ».*

*-« Worked with surgeons I did not know. Relationship created in these exceptional circumstances. We regularly greet each other! ».*

And for some, it led to changes in assignments or professional prospects:

*-« That night, I was in a department among the victims (…); I witnessed and lived through the shortcomings (…) During the weeks that followed, I wanted to express what I felt, what needed to be changed. Since then, I’ve left my job. ».*

*-« Yes, it has changed me, I’m revolted and irritable, and I’m seriously thinking of professional retraining ».*

*-« I am now a reservist at the national gendarmerie, I trained in safety procedures, first aid and defence. ».*

### Use of psychological support

Most of the exposed respondents who answered the question on their use of psychological support resources for exposed health professionals which had been specifically set up, stated that they did not, neither within the hospital or outside the institution, whether in the immediate aftermath of the attack or during the following months (*n* = 275/370, i.e. 74.3%).

Among staff presenting with probable full PTSD, there were significantly more ‘professional exposure only’ staff who did not receive psychological support than ‘bystanders’ (70.6% vs 17.6% *p* = 0.002); the same applies to staff with full or partial PTSD (64.2% vs 42.6%, *p* = 0.025).

Although the differences between these two groups are not statistically significant, the same trends in terms of absence of psychological support were observed among staff members presenting with signs of other mental disturbances: probable state of anxiety (64.3% vs 45%, *p* = 0.184), suicidal thoughts (36.4% vs 11.1%, *p* = 0.319).

For the final items of the web-questionnaire, few exposed professionals (*n* = 52/488) answered the questions exploring the reasons for not using the psychological support resource, so that no specific insight into this issue was provided.

## Discussion

### Main results

From 11 to 15 months after the 2016 Nice terror attack, hospital staff with ‘professional exposure only’ still displayed signs of a significant impact on their mental health: the prevalence rate for full PTSD was 9.4%,, and it was 27.1% for probable full or partial PTSD. Moreover, suicidal thoughts among exposed staff members were significantly more frequent than among the unexposed group. In spite of these alarming signs, nearly three quarters of exposed staff members stated they had not received psychological support.

Full PTSD among staff with ‘professional exposure only’ reached 9.4%, a higher proportion than that observed among the general population over the previous 12 months, i.e. approximately 1% [29, 30]. It is also higher than in the aftermath of the Paris attack of January 2015, estimated at 3% among rescue workers [14] and 4.4% among health professionals following the November 2015 Paris attack [15]. The difference may be explained by the fact that the vast majority of hospital personnel do not work in emergency facilities. Staff members in this study were not only rescue or emergency workers but reflect the whole range of professions involved in the running of a hospital. Moreover, staff with ‘professional exposure only’ were exposed for over 3 weeks, which appears to have seldom been the case in post-terror attack interventions [31]. Our study provides supplementary information on partial PTSD prevalence rate, i.e. 17.7%. Overall, 27% of staff with ‘professional exposure only’ presented with full or partial PTSD. By comparison, the rate was 18.8% among « utility workers » working on the World Trade Center site [31].

In the present study, the risk of developing PTSD among staff with ‘professional exposure only’ can be related to the iterative or extreme exposure to aversive details of traumatic events [9]. It was particularly significant among women, as reported in the literature [1, 13, 32], among persons living in social isolation [15, 33], and among those who, following the attack, were confronted with ‘unfamiliar tasks’, while low predictability in working conditions has been shown to result in psychological distress [34].

Suicidal thoughts were reported by 5.5% of staff with ‘professional exposure only’, vs 1.2% among unexposed staff, *p* = 0.02. This has been observed among firefighters and ambulance staff [35] and our results are close to those identified among Norwegian ambulance personnel [36]. Moreover, the relationship between PTSD and suicidal thoughts has previously been observed among first responders [35], urging monitoring of this population.

These results are particularly worrisome in the hospital setting as symptoms may impact the staff’s professional activity, while, paradoxically, mental health support was underutilized. Indeed, approximately 10% of staff with‘professional exposure only’ went on sick leave or were declared victims of an occupational accident. Six months after the January 2015 Paris attacks, 6% of rescue workers were still unable to return to work [14]. Risks are documented of absenteeism or turnover of stressed personnel, or even of their leaving the institution [37, 38]. Many staff with ‘professional exposure only’ in the present study reported difficulty to concentrate, sleep disorders, fatigue; these symptoms can combine with PTSD and lead to professional difficulties [39], with performance deficits [40] that can in turn compromise quality of patient care. The open comments substantiated the quantitative data, complementing the results by providing a personal perspective of general situations and of the staff’s experience and perceptions.

Nevertheless, 74% of all exposed staff stated they had not received psychological support, a situation which has been observed elsewhere [14, 21]. This is markedly more frequent among staff with ‘professional exposure only’ than among staff with ‘bystander’ exposure who may have felt more justified in seeking psychological support; and even more so among those with full or partial PTSD symptoms, anxiety, or suicidal thoughts. Although reluctance to seek support is well documented among physicians [41], in the current survey this encompasses a broader professional range. We can only speculate on the lack of recourse to psychological support among exposed staff: the setup of the psychological support resources provided by the hospital may not have been suited to staff expectations for various reasons; identification of staff members potentially exposed to a traumatic experience was not considered, hence the lack of a systematic approach to reach out to them and offer them a planned support meeting; on a more individual level, there may have been a fear of a lack of confidentiality when contacting a psychological support specialist in their own workplace [42]. These findings point to the need to dedicate adequate resources to organise early mental health support for exposed hospital staff, and to strongly recommend follow-up which can take on various forms [43].

### Strengths and limitations

While this study’s main focus is the mental health of staff with ‘professional exposure only’, it is not restricted to medical care-related staff. Very few studies have explored the consequences of terror attacks on all members of hospital staff [44–46]. Thus, although publications reporting care-related interventions in Nice University and Lenval hospitals involve a wide array of professions having worked in the institutions in this context [17–20, 47–55], they provide a restricted view as all members of staff were involved in activities required by this exceptional situation, including those least visible [56].

Furthermore, few studies focus on tasks performed by hospital staff in the aftermath of a terror attack. Publications relating the WTC attack are informative in this respect as, while very different from our study population, they describe a wide variety of roles and tasks among rescue and recovery workers according to the professions exposed on the site and the enormity of the experience [57]. For those who worked on the site, having seen bodies or body parts constituted a risk factor for PTSD [31]. For physicians, having had to inform families of a person’s death, or to care for an individual after death is a cause of trauma. Such findings have been confirmed in the Nice context where exposure to morbid details occurred during professional activity [33], especially as, to our knowledge, few terror attacks in Europe have resulted in so many child and adolescent victims: 40% of staff with ‘professional exposure only’ in the present study took part in the management of young patients, although only 21% worked in a paediatric department at the time of the attack.

The open comments substantiated the quantitative data, complementing the results by providing a personal perspective of general situations and of the staff’s experience and perceptions.

The study has several limitations. Since it was impossible to identify hospital staff who had been directly or indirectly exposed to the attack, the survey had to cover all staff members, including those unexposed. Even using various and sustained communication procedures during the extent of the study period, the participation rate for the study was only 8%, i.e. far lower than expected. Compared to those non-respondents who were not physicians, among non-medical personnel who participated in our survey, women were over-represented, as well as management, administrative and medico-technical staff. However, as these professional categories are not caregivers, they are professionally less exposed to injured or deceased casualties or accounts by victims, which may have decreased the observed mental health impact.

Research on terror attacks involving web questionnaires is known to encounter difficulties in accessing trauma-experienced populations, raising the issue of representativeness [58]. Moreover, few studies report the participation rate of exposed professionals following a terror attack. By comparison, a study revealed that the participation rate was 25% among firefighters in Paris and affiliated volunteers following the November 2015 Paris attack; but this rate could not be assessed and was probably much lower among health professionals [15]. This rate may have resulted in bias, as discussed elsewhere [23]. Indeed, unexposed personnel may have felt unconcerned by the survey and not considered it worthwhile to participate: thus, only 1014 among all the employees chose to connect to the web questionnaire, and 804 completed it, i.e. a 79% response rate. This reflects a self-selection bias, in which individuals are more likely to respond to items which interest them or which are directly related to their own situation [59]; however, the most members of the hospital staff were probably unexposed. Conversely, those most exposed may have refused to complete the questionnaire to avoid recalling painful memories [60], while studies insist on the need to improve participation to identify individuals with the highest morbidity rate [61]. Moreover, staff members who might have left the institutions after the attacks were not able to take part in the survey and experiencing this event may have increased staff turnover and resulted in staff leaving the institution, so that the prevalence rates are likely to be underestimated.

### Implications

Despite these limitations, the present study, conducted after the Nice terror attack, provides a useful overview of hospital staff’s mental health when exclusively exposed through their professional activity, even those whose jobs did not normally involve clinical care. Their comments reveal the strength of the impact of such a type of exposure. Although the Nice Hospital administrations had set up resources following the attack to assess the potential health impact and to offer staff members psychological support [23], our results show that this approach did not have the expected result, few staff with ‘professional exposure only’ sought psychological support, especially among those experiencing the most symptoms.

In order to improve the health of exposed hospital staff, authors have also recommended developing their capacity for psychological resilience [62], emphasizing the role of such factors as a supportive hierarchy [33], prior training on the risk of PTSD [15], their personal experience and feeling of competence, all of which may participate in the development of post-traumatic growth [63]. Programmes designed to anticipate, plan and deter risks confronted by staff exposed to catastrophic events could thus offer alternative approaches to manage and pro-actively mitigate professional risk factors [64, 65]. At a time when the current pandemic exerts a major strain on health institutions’ human resources, robust prevention and follow-up programs must be developed for hospital staff, in order to manage the health risks they face when exposed to exceptional health-related events such as mass terror attacks.

## Data Availability

Data are available upon request. Contact person: Roxane FABRE (fabre.r@chu-nice.fr).

## Abbreviations

HAD: Hospital anxiety and depression scale
PTSD: Post-traumatic stress disorder
CHU: Centre Hospitalo-Universitaire de Nice (Nice University Hospital)
